# ProtSA: a web application for calculating sequence specific protein solvent accessibilities in the unfolded ensemble

**DOI:** 10.1186/1471-2105-10-104

**Published:** 2009-04-08

**Authors:** Jorge Estrada, Pau Bernadó, Martin Blackledge, Javier Sancho

**Affiliations:** 1 Departamento de Bioquímica y Biología Molecular y Celular, Facultad de Ciencias, Universidad de Zaragoza, 50009 Zaragoza, Spain; 2 Biocomputation and Physics of Complex Systems Institute (BIFI), Universidad de Zaragoza, 50009 Zaragoza, Spain; 3 Biomolecular NMR, Institute for Research in Biomedicine, Parc Científic de Barcelona, 08028 Barcelona, Spain; 4 Institut de Biologie Structurale Jean-Pierre Ebel, 38027 Grenoble, France

## Abstract

**Background:**

The stability of proteins is governed by the heat capacity, enthalpy and entropy changes of folding, which are strongly correlated to the change in solvent accessible surface area experienced by the polypeptide. While the surface exposed in the folded state can be easily determined, accessibilities for the unfolded state at the atomic level cannot be obtained experimentally and are typically estimated using simplistic models of the unfolded ensemble. A web application providing realistic accessibilities of the unfolded ensemble of a given protein at the atomic level will prove useful.

**Results:**

ProtSA, a web application that calculates sequence-specific solvent accessibilities of the unfolded state ensembles of proteins has been developed and made freely available to the scientific community. The input is the amino acid sequence of the protein of interest. ProtSA follows a previously published calculation protocol which uses the Flexible-Meccano algorithm to generate unfolded conformations representative of the unfolded ensemble of the protein, and uses the exact analytical software ALPHASURF to calculate atom solvent accessibilities, which are averaged on the ensemble.

**Conclusion:**

ProtSA is a novel tool for the researcher investigating protein folding energetics. The sequence specific atom accessibilities provided by ProtSA will allow obtaining better estimates of the contribution of the hydrophobic effect to the free energy of folding, will help to refine existing parameterizations of protein folding energetics, and will be useful to understand the influence of point mutations on protein stability.

## Background

A detailed understanding of protein folding energetics is fundamental for *ab initio *prediction of protein 3-D structures from sequences, for the rational engineering of new proteins, and for understanding diseases related to protein misfolding or aggregation [1,2]. The unfolded state of proteins is central in developing the theoretical framework of folding processes because it represents the starting point from which proteins evolve to the native state. The hydrophobic effect operating on apolar side chains is an important factor driving protein folding [1], and the change in solvent accessible surface area (SASA) of a protein upon folding can be used to estimate the contribution of the hydrophobic effect to the free energy of folding [1,3]. Empirical models relate changes in SASA (total, polar or apolar) upon folding to the heat capacity, enthalpy, or entropy of folding, and to equilibrium m-values in chemical unfolding [4,5].

The SASA of a protein was defined [6] as the surface described around the protein by the centre of a solvent sphere in contact with the van der Waals surface of the molecule. Experimental determination of accurate SASAs of folded proteins at the atom level is not yet possible. Fortunately, computation of SASA values in the native state is straightforward when 3-D structures are available. Accurate SASA values for the unfolded state are not only difficult to determine but also difficult to calculate. In attempts to calculate the changes in SASA associated to the protein folding reaction, a variety of models of the unfolded state have been proposed. They include tripeptides [7-9], peptide-fragment collections in both native and extended conformations, extracted from a set of native structures [10,11], ensembles of Ac-(Ala)_3_-X-(Ala)_3_-Nme peptides [12], and ensembles of polypeptide conformations of a specific selected protein [13]. A common characteristic of all these models, but the last one, is that they provide mean solvent accessibilities for the 20 residue types, but they do not take into account the possibility that these accessibilities are modulated by the specific sequence context of the residue of interest.

We have recently developed a way to estimate SASA at atomic resolution in the unfolded ensemble. The method provides individual SASAs for each atom of each residue in a given protein sequence [14]. The structural model chosen to describe the unfolded state consists of hundreds to thousands of unfolded conformations generated by Flexible-Meccano, an algorithm that performs conformational sampling using a coil-library and a simple volume exclusion term [15]. The ensembles generated in this way successfully describe backbone fluctuations of several intrinsically unfolded proteins probed by Nuclear Magnetic Resonance (NMR) and small-angle X-ray scattering (SAXS) [15-19]. Our analysis of solvent exposures in unfolded ensembles of proteins generated with this method clearly indicates that the SASA of any residue is strongly influenced by its sequence neighbours [14] and, therefore, using generic residue-type values is not justified. A detailed benchmarking of the method has been described [14].

Here, we present a web application that calculates SASA of protein unfolded-state ensembles, detailed per residue and atom, using the methodology described [14]. As far as we know, only two related servers exist. *BPPred *[20] calculates, from the number of residues, an overall protein change of SASA upon folding. *Unfolded *implements the approach by [11], which is based in generic residue-type values. None of these two servers calculates SASA values on a sequence specific representation of the unfolded-state ensemble of the protein of interest. In this sense, ProtSA is an innovative web application that will provide researchers with more accurate accessibility data for the parameterization and interpretation of protein folding thermodynamics.

## Implementation

ProtSA architecture consists of three parts: the user web browser, a middle tier Common Gateway Interface (CGI) application running on a web server, and the server part that calculates SASA of the protein unfolded-state ensemble. The server part uses three external software programs to perform the calculations: Flexible-Meccano for backbone-conformation generation, SCCOMP[21] for side chain building, and ALPHASURF[22] for SASA calculations of each conformation of the unfolded ensemble of the requested protein. The interaction between the ProtSA parts is as follows: the user fills in the input form using the web browser; the browser sends the input data to the CGI application, which checks its completeness and validity, and redirects complete and valid requests to the server part; the server part is a multithreaded program, with one network thread for receiving requests, and several worker threads for processing requests (one request per thread); the network thread receives the request from the CGI application, checks for resource availability and replies to the CGI application with an acceptance or refusal message; the CGI application informs the user whether the request is accepted or not, with the reason for refusal in the latter case; if resources are available, the network thread in the server part queues the request and, when a worker thread becomes available and no earlier requests are queued, that worker thread processes the request, calculating SASA of the protein unfolded-state ensemble; finally, the worker thread emails the results to the user. Both the CGI application and the server part were programmed using C++.

ProtSA basically follows the method shown in [14] for calculating SASA of a protein unfolded-state ensemble, though ProtSA uses ALPHASURF instead of NACCESS[23] for the calculations of each unfolded-state protein conformation. ALPHASURF was chosen because it uses an exact analytical method (based on the alpha shape theory) and is free software; the results section shows that ALPHASURF and NACCESS give very similar results. The steps of the ProtSA method are:

1. Check that all residues in the protein sequence belong to the set of 20 standard types. If the user provides a 3-D structure, check it for gaps or missing atoms.

2. Generate, from the protein sequence, a set of unfolded-state backbone-only conformations using Flexible-Meccano.

3. Add side chains to each conformation using SCCOMP.

4. Calculate SASA of each conformation using ALPHASURF. Obtain mean values per residue and per atom.

5. (Only if the user provides a 3-D structure of the protein) Calculate SASA for the 3-D structure (assumed to represent the folded state) using ALPHASURF. Calculate differences between folded and unfolded SASA, per atom and per residue.

Flexible-Meccano's Monte Carlo algorithm for generating the backbone of the unfolded-state conformations uses a subset of the database of amino-acid-specific Φ-and Ψ-torsion angles described in [24]; the subset is obtained by exclusion of all residues in α-helices and β-sheets. The database includes symmetric values for glycine Φ- and Ψ-torsion angles, and has special cases for residues preceding a proline. For each protein unfolded-state conformation the algorithm constructs the backbone starting at the C-terminal, although it has been shown that building directionality does not influence SASA results [14]. Residue *i *is connected to residue *i+1 *by selecting a random pair of Φ- and Ψ-angles, for the type of residue *i*, from the torsional subset database. If residue *i *presents clashes with other residues (where residues are represented as spheres centred at the Cβ atom -the Cα atom for glycine residues- using radii derived from Levitt's force-field [25]), the Φ- and Ψ-torsion-angle pair is rejected, and another one is randomly selected. If, after 500 tries, the algorithm does not find a non-clashing Φ-and Ψ-torsion-angle pair, the partially-built conformation is rejected and the algorithm starts again at the C-terminal residue.

A key factor to the sequence-specificity of SASAs calculated for unfolded ensembles is the decoration of each polypeptide backbone with energetically realistic conformers of the sequence residues. This is performed using the iterative method implemented in SCCOMP. Using the rotamers of a backbone-dependent library, and a backbone independent one for special locations in the protein chain (such as the first and last residues), SCCOMP assigns rotamers, residue by residue, optimizing a scoring function with terms accounting for atom-atom contacts, steric overlaps, torsion energy, and the hydrophobic effect. SCCOMP repeats the complete assignment of rotamers to the protein residues until either there is no change in structure in two consecutive iterations or the limit of allowed iterations is reached.

## Results

The original method described to calculate solvent accessibilities in unfolded ensembles [14] used NACCESS, while the ProtSA application relies in ALPHASURF. We have compared the performance of these two methods by recalculating unfolded solvent accessibilities for the set of 19 proteins used in the original implementation. Another popular program to calculate exposures, DSSP [26], yields values 5% higher than those of NACCESS and ALPHASURF (not shown).

The results of the new calculations performed with ALPHASURF are shown in Table 1 compared with those obtained with NACCESS and previously reported [14]. The two algorithms provide very similar exposures for the same protein with overall SASA values differing less than 0.36%. The average, minimum and maximum SASA accessibilities found for each residue type within the unfolded ensembles of the 19 proteins are shown in Table 2. Differences with the original data reported [14] are also minimal. For average residue SASAs, the biggest difference (0.53%) is for methionine (Table 2 and data in [14]), and the mean of the differences observed for all the residues using the two methods is 0.19%. Similarly, for the minimum value of SASA found for each residue type within the 19 ensembles, the biggest difference is at 2.04% for one specific threonine residue, with a mean of 0.77% for the twenty residue types. For the maximum SASA values for residue types, the biggest difference is 3.15% for one specific glutamic acid residue (mean difference of all maximally exposed residues being 0.81%). The main utility of ProtSA calculations is that they can highlight strong divergences in the exposure of specific residues from the average values exhibited by their corresponding residue types in the unfolded ensemble (Table 2). These divergences are sequence context dependent and can only be revealed with sequence specific calculations.

**Table 1:** ProtSA solvent accessibilities of unfolded ensembles of test proteins

Protein PDB code	Number of residues	Accessibilities by ProtSA^1^	Accessibilities by NACCESS^2^
[PDB:1LN4]	98	10520	10497
[PDB:1T1D]	100	11170	11136
[PDB:1BKR]	109	11683	11666
[PDB:1BGF]	124	13843	13808
[PDB:1JB3]	131	14300	14261
[PDB:2LIS]	136	15320	15302
[PDB:1QGV]	142	15648	15632
[PDB:1EY4]	149	16437	16430
[PDB:1EP0]	185	20568	20529
[PDB:1L3K]	196	21442	21436
[PDB:1BYI]	224	23476	23393
[PDB:1ES9]	232	25251	25250
[PDB:1II5]	233	25330	25258
[PDB:1WER]	334	37937	37845
[PDB:1FO9]	348	39539	39492
[PDB:1FCQ]	350	39941	39877
[PDB:1E5M]	416	43551	43429
[PDB:1GSO]	431	45765	45628
[PDB:2BCE]	579	62744	62631

^1^Solvent accessibilities (in Å^2^) calculated with ProtSA, using ALPHASURF. They are averaged over 2000 unfolded structures of each protein. The first and the last five residues of each sequence are not taken into account.

^2^Solvent accessibilities (in Å^2^) calculated with NACCESS in otherwise identical conditions (taken from Table 3 in reference [14]).

**Table 2:** Solvent accessibilities (Å^2^) of amino acid residues in protein unfolded ensembles calculated with ProtSA

Residue	Number of Residues^1^	Average^2^	Minimum^3^	Maximum^4^	% Difference^5^
Ala	349	73.2 (73.1)	58.2	84.2	31
Arg	233	178.9 (178.6)	155.3	192.8	19
Asn	198	109.2 (109.1)	91.1	121.5	25
Asp	255	102.2 (102.0)	83.8	117.2	29
Cys	51	88.7 (88.3)	76.7	98.2	22
Glu	287	126.0 (125.9)	108.9	140.9	23
Gln	171	125.9 (125.6)	108.6	141.6	23
Gly	312	54.3 (54.2)	36.6	65.6	44
His	115	129.5 (129.3)	109.0	140.0	22
Ile	229	122.5 (122.2)	107.2	135.5	21
Leu	407	131.9 (131.5)	110.3	147.7	25
Lys	247	149.9 (149.8)	131.2	167.2	22
Met	102	134.3 (133.6)	122.0	149.1	18
Phe	174	146.1 (146.1)	130.7	163.2	20
Pro	217	100.3 (100.0)	81.8	123.4	34
Ser	198	76.0 (75.8)	59.3	90.5	34
Thr	245	93.3 (93.2)	79.7	107.6	26
Trp	70	173.2 (173.0)	161.8	185.1	13
Tyr	148	156.9 (156.8)	140.1	173.2	19
Val	319	102.2 (102.0)	84.8	115.3	26

Mean		118.7 (118.5)	101.9	133.0	25

^1^Total number of residues of that kind found in the 19 protein sequences simulated.

^2^Residue-specific solvent exposure averages. The numbers in parenthesis were calculated with NACCESS (see [14])

^3^Minimum solvent exposure found in one of the 19 denatured ensembles.

^4^Maximum solvent exposure found in one of the 19 denatured ensembles.

^5^Percentage difference between maximum and minimum solvent exposures found for one residue type: 100(max-min)/max

**Figure 1:**
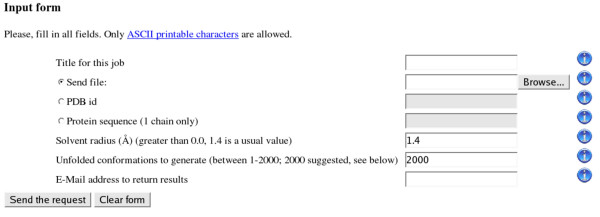
**Input form of the ProtSA web application**. Through this simple input form the user can identify the request with a title, and submit the protein information in three different ways (as a PDB file, as a PDB id, or as a chain of residues in text form). The user sets the solvent radius and the number of unfolded conformations to generate for each chain in the protein. ProtSA sends the results of the calculations to the email address of the user.

ProtSA is available at [27]. The input web form in ProtSA is very simple (Fig. 1). The user can supply a protein sequence, a PDB-formatted file, or a PDB id. The user must also specify the number of protein conformations to generate, and the radius of the solvent probe. Specification of probe radius may be used for calculating surface accessibility to different ions, not just water molecules. The user also specifies the email address where ProtSA will mail the results. When the user supplies a protein sequence (which must be a single-chain one), ProtSA calculates only the SASA for the unfolded ensemble. When the user supplies a PDB file or a PDB id (which ProtSA uses to fetch the corresponding PDB file from the Protein Data Bank [28]), ProtSA also calculates the SASA for the 3-D structure, which is assumed to represent the folded state. ProtSA emails the user the calculated results. For each atom and residue, the results include the average sequence-specific SASA in the unfolded ensemble and, if it was calculated, the SASA in the folded state, and the difference (SASA_folded _– SASA_unfolded_).

**Figure 2:**
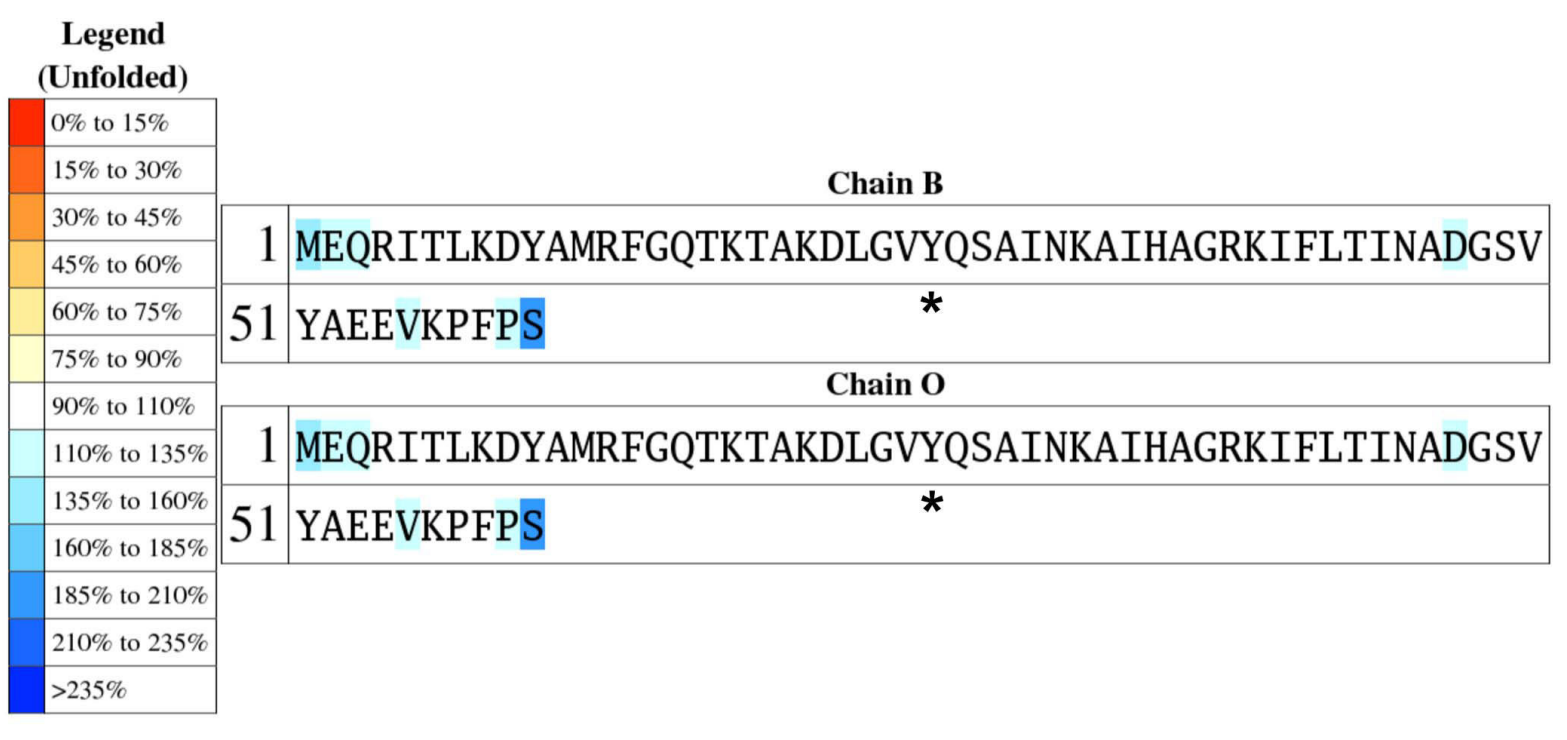
**Colour-coded sequence for the unfolded SASA of **[PDB:5cro]**, calculated with ProtSA**. Almost all residues are in the range 90%–110% of the average unfolded SASA for its residue type (from a set of 19 proteins). As expected, residues in the extremes of the chains are more exposed than the average. The data shown is for 1.4 Å of solvent radius and 2000 conformations. Tyr 26 is labelled.

**Figure 3:**
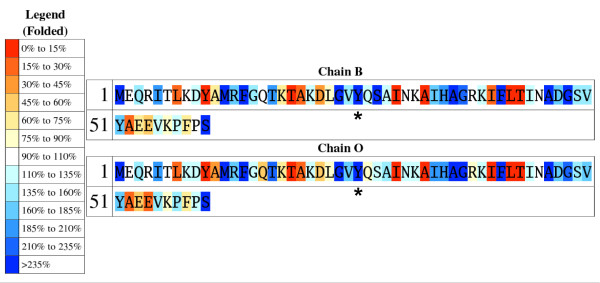
**Colour-coded sequence for the folded SASA of **[PDB:5cro]**, calculated with ProtSA**. The specific degree of burial in the folded state of a given residue type varies widely. Tyr 26 is clearly more exposed than the average tyrosine residues present in the test set of 11 proteins (see text), which could be suggestive of a possible 'reverse hydrophobic effect'. This effect can be discarded by inspection of figure 4a. The data shown is for 1.4 Å of solvent radius and 2000 conformations. Tyr 26 is labelled.

**Figure 4:**
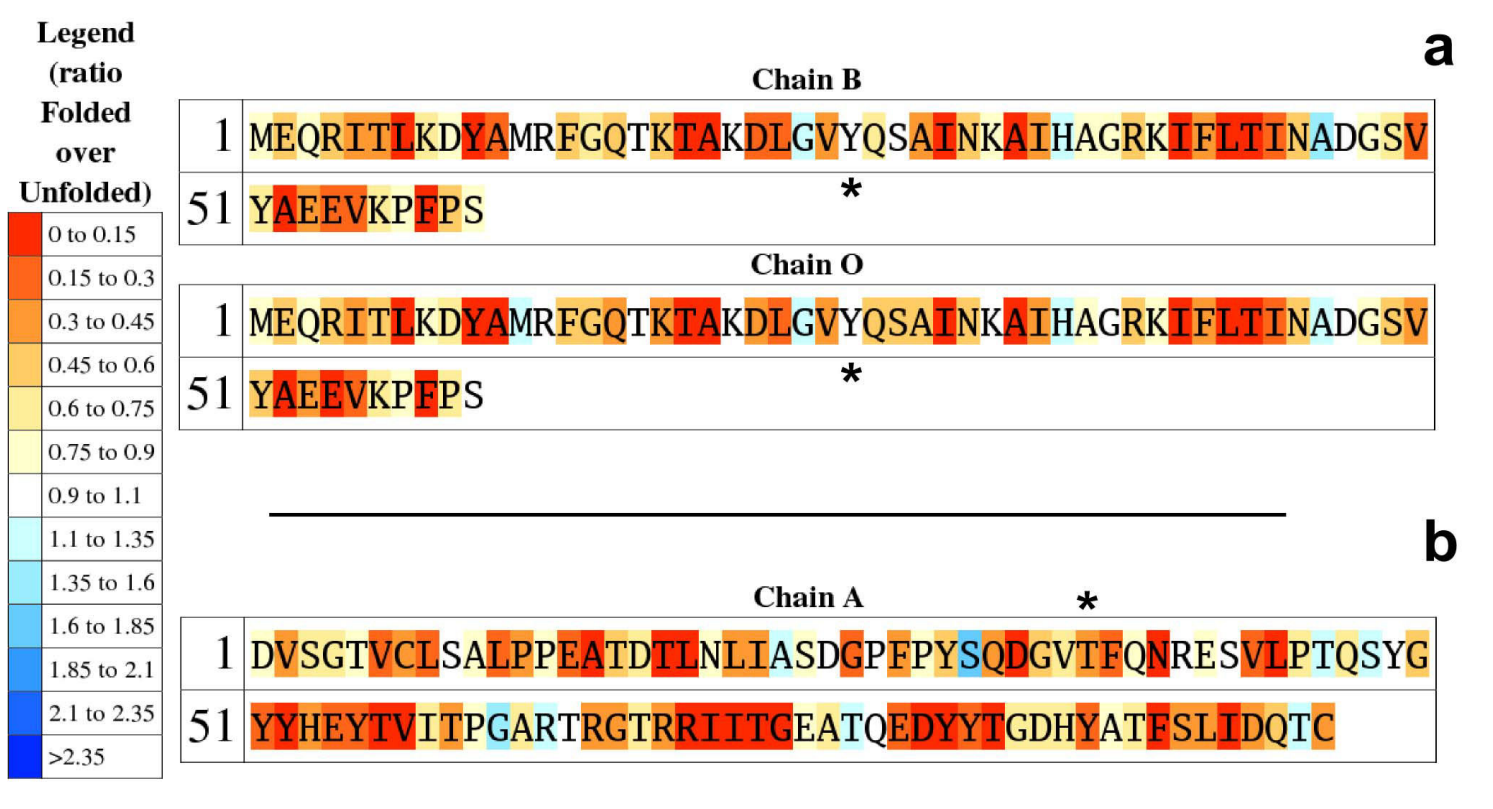
**Colour-coded sequences of the ratio of folded and unfolded SASA for **[PDB:5cro]** and **[PDB:1ucj]. Upon folding, most residues tend to be less exposed to solvent than in the unfolded state, as shown by the majority of reddish residues in the figure. However, some residues may be more exposed in the folded state (bluish residues). a) Tyr 26 (labelled) of [PDB:5cro] has a ratio of folded SASA over unfolded SASA in the range 0.9–1.1, suggesting that no reverse hydrophobic effect is taking place (see text). b) Thr 36 (labelled) of [PDB:1ucj], as most residues, is also in a reddish shade. Since this residue bears a polar side chain, its burial is suggestive of a destabilizing contribution to the native conformation. The data shown in both cases is for 1.4 Å of solvent radius and 2000 conformations.

**Figure 5:**
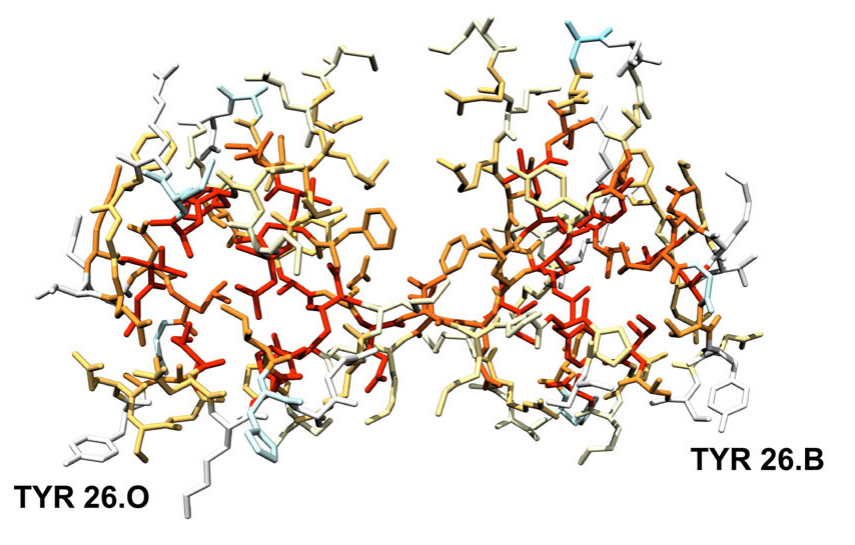
**X-ray structure of **[PDB:5cro]** colour-coded by the ratio of folded SASA over unfolded SASA**. Colour-code is as in the legend of figure 4. Tyr 26, labelled, appears in white colour, indicating that its solvent exposure in the folded state is very similar to that in the unfolded ensemble.

To highlight those residues with unusually high or low exposures in the unfolded ensemble relative to typical values (calculated as the average exposures in the 19 test proteins), the protein sequence is also returned colour-coded so that underexposed residues appear in a gradation of red colours and overexposed ones in a gradation of blue colours (*unfolded sequence*; Fig. 2). This plot allows detecting residues that are more exposed than expected, either because they appear in terminal regions, or because they are surrounded by small residues. When ProtSA calculates SASAs for the folded state, two additional colour-coded protein sequences are returned. One of them (*folded sequence*; Fig. 3) depicts residues comparing their folded SASA values to those of the average folded SASA of the 20 residue types in an 11-protein subset of the 19 test-sequences (those with all their atoms present in the folded structure). The other one (*ratio sequence; *Fig. 4) depicts, for each residue, the ratio between its folded SASA and its unfolded SASA. In addition, the original PDB file is returned with those SASA ratios replacing the B-factors, to allow a straightforward three-dimensional visualization of exposure changes associated to protein folding (Fig. 5). Two examples illustrate the usefulness of these coloured sequences for pinpointing residues that may significantly contribute to protein stability as a consequence of unusually high or low solvent exposures. The first one refers to a classical 1990 article [29] where a reverse hydrophobic effect on Tyr 26 of the λ-cro protein was proposed, based upon an estimated 1.4-fold hyper exposition in the folded state compared to that in the unfolded state. Subsequent studies refuted this proposal by showing that the ratio was close to 1.0 [30]. ProtSA, using a more detailed model of the unfolded state, clearly supports the latter studies. It only requires the user a single look at the coloured *ratio sequence *(Fig. 4a) where Tyr 26 appears on a white background, to grasp that the folded-to-unfolded ratio is close to 1.0. On the other hand, inspection of the *folded sequence *(figure 3) reveals that the exposure of this tyrosine residue in the folded state is well above average, which could have influenced the initial interpretation. The second example refers to Thr 36 in the V36T mutant of RNase Sa. Compared to the wild type protein, the presence of a threonine at position 36 destabilizes the folded state [31]. ProtSA depicts Thr 36 in the *ratio sequence *on an orange background (Fig. 4b), which visually indicates this residue losses a large percentage of its solvent exposure upon folding, and is expected to destabilize the folded conformation. Incidentally, the *ratio sequence *for this protein shows that additional polar residues appear more buried in the folded conformation than in the unfolded ensemble. To asses whether they are likely to destabilize the native conformation their local environments should be analysed. They may establish appropriate compensating polar interactions or else they should be considered as potentially destabilising residues.

From the execution times of the 19 test proteins in a computer running CentOS 5, with 2 GB of RAM and a Core 2 Duo-2.4 GHz CPU (data not shown) we can deduce a linear dependence on the size of the input sequence. For the more demanding requests corresponding to ensembles of 2000 protein molecules there is a fix cost of about 80 minutes and a variable cost of about 0.5 minutes per residue in the sequence.

## Conclusion

ProtSA, the freely-available web application presented in this work, represents a novel tool for the researcher interested in protein folding energetics. The sequence-specific protein solvent accessibilities in the unfolded state ensemble calculated by ProtSA will provide researchers a more precise view of unfolded state ensembles, and will help to understand the influence of mutations on protein stability.

## Availability and requirements

Project name: ProtSA

Project home page: 

Operating system(s): Platform independent

Programming language: C/C++

Other requirements: JavaScript-enabled web browser

Any restrictions to use by non-academics: None

## Authors' contributions

JE developed ProtSA. PB and JS directed the design. MB created software parts. All authors wrote the manuscript, and read and approved the final version.
